# Molecular modeling provides a structural basis for PERK inhibitor selectivity towards RIPK1†‡

**DOI:** 10.1039/c9ra08047c

**Published:** 2020-01-02

**Authors:** Chetan Chintha, Antonio Carlesso, Adrienne M. Gorman, Afshin Samali, Leif A. Eriksson

**Affiliations:** Apoptosis Research Centre, National University of Ireland Galway Galway Ireland; Department of Chemistry and Molecular Biology, University of Gothenburg 405 30 Göteborg Sweden leif.eriksson@chem.gu.se

## Abstract

Protein kinases are crucial drug targets in cancer therapy. Kinase inhibitors are promiscuous in nature due to the highly conserved nature of the kinase ATP binding pockets. PERK has emerged as a potential therapeutic target in cancer. However, PERK inhibitors GSK2606414 and GSK2656157 also target RIPK1 whereas AMG44 is more specific to PERK. To understand the structural basis for the selectivity of PERK ligands to RIPK1 we have undertaken a detailed *in silico* analysis using molecular docking followed by molecular dynamics simulations to explore the selectivity profiles of the compounds. Although the binding sites of PERK and RIPK1 are similar, their binding response to small molecules is different. The docking models revealed a common binding mode for GSK2606414 and GSK2656157 in the RIPK1 binding site, similar to its cognate ligand. In contrast, AMG44 had a strikingly different predicted binding profile in the RIPK1 binding site with both rigid docking and induced fit docking settings. Our study shows a molecular mechanism responsible for dual targeting by the GSK ligands. More broadly, this work illustrates the potential of molecular docking to correctly predict the binding towards different kinase structures, and will aid in the design of selective PERK kinase inhibitors.

## Abstract 2

Molecular modelling explains the lack of selectivity for inhibitors GSK2606414 and GSK2656157, as compared to inhibitor AMG44.

## Graphical abstract

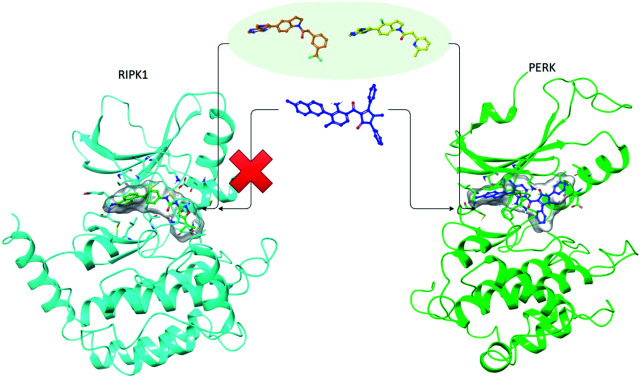

## 1. Introduction

Kinase inhibitors often display a high degree of promiscuity due to the structural similarity of their ATP binding sites.^1^ Large scale *in vitro* binding experiments have identified several previously unknown off-target kinase interactions,^2,3^ and small molecules that selectively target a kinase within a subfamily can have off-targets in a different family. The selectivity profile can thus limit the clinical applications of an inhibitor, and uncharacterized off-target effects often lead to toxicity. Lack of understanding of selectivity can lead to misinterpretation of preclinical and clinical outcomes. In the current study, we investigate the cross-reactivity of Protein kinase RNA-like endoplasmic reticulum kinase (PERK) inhibitors. PERK, activating transcription factor 6 (ATF6) and inositol-requiring enzyme 1α (IRE1) are the three main endoplasmic reticulum (ER) resident transmembrane proteins that sense endoplasmic reticulum (ER) stress levels and initiate the unfolded protein response (UPR). PERK is a eukaryotic initiation factor 2 alpha (eIF2α) kinase. Upon activation, PERK's cytoplasmic kinase domain dimerizes initiating *trans*-autophosphorylation of the activation loop and subsequent phosphorylation of eIF2α at Ser51.^4^ Phosphorylated eIF2α (P-eIF2α) binds to eIF2B resulting in a shutdown of global protein synthesis which in turn reduces the protein load to the ER.

Pharmacological PERK inhibition has been shown to reduce tumor growth in mouse xenograft models.^5,6^ PERK inhibition is also proven beneficial in neurodegenerative conditions such as prion disease, frontotemporal dementia and Parkinson's disease.^7,8^ Prominent PERK inhibitors include ATP-competitive kinase inhibitors GSK2606414 (GSK414),^5^ GSK2656157 (GSK157)^5^ and Compound 44 from Amgen (AMG44)^9^ amongst others.^10^ GSK414 and GSK517 are selective with respect to other eIF2α kinases such as PKR, GCN2, HRI and also have good overall kinome-wide selectivity.^5^ However, both GSK414 and GSK157 compounds were recently shown to inhibit receptor-interacting kinase 1 (RIPK1) with nanomolar potency.^11^ RIPK1 is involved in the TNFα mediated inflammatory signaling pathway and necroptosis.^12^ Independently, GSK414 was shown to also inhibit tyrosine kinase receptor c-KIT.^13^ Interestingly, the structurally different PERK inhibitor AMG44 does not appear to inhibit RIPK1 or c-KIT.^11,13^ Hence, AMG44 is proposed to be a better tool compound to probe for PERK signaling despite the lack of tissue distribution data of the compound. On the other hand, GSK414 crosses the blood-brain barrier (BBB) when orally delivered, making it a better choice of inhibitor for central nervous system (CNS) applications.^7^ Clearly, selective PERK inhibitors that can cross BBB will have a wide range of clinical applications.

With the aim of understanding PERK inhibitor cross-reactivity, we analyzed the binding profiles of GSK414, GSK157, and AMG44 in the RIPK1 active site. We used computational modeling methods to compare the protein-ligand interaction fingerprints between the two targets. We use molecular docking and molecular dynamics (MD) simulations for analyzing the binding modes of the compounds. Docking methods have been widely used in virtual screening for kinase targets and kinase drug repurposing.^14–16^ MD simulations provide atomistic insight into the dynamic behavior of protein-ligand complexes as a function of time.^17^ Rationalizing the selectivity profile with differences in binding modes will enable the design of selective PERK inhibitors. Despite large-scale kinome-wide screens used for selectivity profiling, potential off-targets are often not detected or underestimated. As an alternative strategy we propose to employ computational methods to perceive selectivity using available structural data.

## 2. Methods

### 2.1. Selection and preparation of protein structure and ligands

The co-crystallized structures of PERK in complex with GSK414 (PDB 4G31), GSK157 (PDB 4M7I) and AMG44 (4X7N), and RIPK1 in complex with Compound 8 (PDB 4NEU), were downloaded from the protein data bank.^18^ The structures were superposed and binding sites were visually inspected in Maestro.^19^ Selected structures were prepared using Schrödinger protein preparation wizard.^20^ Briefly, the protein structures were analyzed for missing loops and added if necessary using Prime,^21^ followed by addition of hydrogen atoms, protonation, and generation of tautomeric states (for Asp, Glu, Arg, Lys and His) for pH of 7.2. Schrodinger OPLS3 force field^22^ was used for protein-energy minimization.

Small molecules were prepared using LigPrep.^23^ Hydrogen atoms were added and different protonation states and ionization states for each ligand were generated for a pH range of 7 ± 2. All possible stereoisomers and tautomeric states were also generated. Finally, the OPLS3 force field^22^ was used for geometry optimization and energy minimization to generate low energy 3D conformers of the ligands.

### 2.2. Identification of RIPK1 similar binding sites

The ProBis server^24^ was used to identify structurally similar protein binding sites to the RIPK1 kinase active site, using the RIPK1 crystal structure PDB 4NEU as query. Binding sites with similar geometrical and physicochemical properties were identified by the ProBis algorithm, and the hits ranked using a standardized *Z*-score. The ProBiS *Z*-score is a statistical measure of structural significance of local structural alignments. The protein structures with *Z*-score > 2.0 are considered to be significantly similar to the query.

### 2.3. Key interaction points (KIPs)

The RIPK1 co-crystal structure and docked complexes were used for calculation of KIPs. Individual electrostatic and hydrophobic contributions to the interaction energy of amino acid residues within 5 Å of the ligand were investigated. The electrostatic (kcal mol^−1^) and hydrophobic contributions (score in arbitrary units) were calculated for each complex by using MOE.^25^ The interaction energy patterns are represented as heat maps using gnuplot.^26^

### 2.4. Druggability assessment of the ATP binding site

The SiteMap module^27^ in Schrödinger was used to calculate the binding site properties for the crystal structures of PERK and RIPK1. SiteMap default parameters were used to calculate properties such as the volume of the pocket, the enclosure/exposure, and the degree of hydrophobicity. Initially, the SiteMap algorithm searches over a grid to identify “site points”, from which contour maps (“site maps”) are generated. The hydrophilic, hydrophobic, Dscore and SiteScore properties are calculated by the formulae:Grid_philic = vdW_energy + oriented-dipole_energyGrid_phobic = vdW_energy − 0.30 × oriented-dipole_energyDscore = 0.094 sqrt(*n*) + 0.60*e* − 0.324*p*SiteScore = 0.0733 sqrt(*n*) + 0.6688*e* − 0.20*p*where *n* is the number of site points (capped at 100), *e* is the enclosure score, and *p* is the hydrophilic score (capped at 1.0). SiteScore > 0.80 is an indicative for promising drug-binding site.^27^ Dscore or druggability score penalizes the increasing hydrophilicity and is thus used as a druggability measure for a pocket. In general, Dscore < 0.83 is considered as “undruggable”, 0.83–0.98 as “difficult to drug” and >0.98 as “druggable”.^27^

### 2.5. Molecular docking

The compounds were docked using Glide^28^ program using default settings unless specified. The OPLS3 force field^22^ was used for the docking protocol. The prepared protein structures were used for generating receptor grids for the docking, using cubic grids with a side length of 20 Å. No constraints were applied for grid generation. The grid center was set at the centroid of the bound ligand in the kinase active site. Prepared ligands were docked using standard precision (SP) mode by enabling flexible ligand sampling for docking procedure. The default parameters of 0.8 scaling factor for van der Waals radii of the nonpolar ligand atoms and 0.15 partial charge cutoff were used. Post-docking minimization was performed on all poses and 20 best conformations for each ligand were saved. The default scoring function Glide Docking Score^28^ was calculated and used for sorting the poses. The chosen docking settings were verified to successfully predict the known crystallographic ligand binding mode of PDB 4NEU.

### 2.6. Induced fit docking

The induced fit docking (IFD) program in the Schrödinger suite was also used in docking studies.^29^ The program combines Glide docking with Prime conformational refinement. Initially, the ligands were docked using a softened potential with Glide SP. The Coulomb-vdW scaling factors were changed to 0.5 for both protein and ligand, and a maximum of 20 poses generated. The generated poses were further processed by Prime for side-chain refinements within 5 Å of the binding site, for better accommodation of the ligands. Thereafter, the systems were minimized with the OPLS3 force field.^22^ In the final step, the ligands were redocked using Glide SP into the optimized protein structures generated within 30 kcal mol^−1^ of the lowest energy structure obtained after Prime refinement to generate 20 poses per system. The poses were ranked using the calculated IFD score (IFDScore = 1.0 × GlideScore + 0.05 × PrimeEnergy), and analyzed manually with the ligand interactions visualized and rendered using Maestro 9.8.^19^

### 2.7. Molecular dynamics simulations

The Desmond program^30^ in Schrödinger suite 2017-2 was used to carry out classical MD simulations for the co-crystal structures and selected docked poses. Each protein-ligand complex was solvated in a simple point charge (SPC) model^31^ using an orthorhombic box with periodic boundary conditions. The overall charge of each system was neutralized by adding Na^+^ or Cl^−^ ions as appropriate. The NPT ensemble available within the Desmond package was used for minimization and relaxation of system. Each simulation was run for a total of 100 ns with a recording interval of 100 ps. The temperature and pressure were kept constant at 300 K and 1.01325 bar, respectively, throughout the simulations. Data analyses such as root mean square deviations (RMSD) and ligand interaction fingerprints were performed using the simulation interaction diagram (SID) program in Schrödinger.

### 2.8. Prime molecular mechanics-generalized born surface area (MMGBSA) calculations

The Prime module in Schrödinger suite 2017-2 was used to compute the ligand binding energies through the use of a physics-based MMGBSA method.^32,33^ MMGBSA free energy of binding (DG bind) is calculated for the docked poses and Desmond trajectories using the equation:^32^DG bind = *E*_Complex_ − *E*_Ligand_ − *E*_Receptor_where *E*_Complex_, *E*_Ligand_, and *E*_Receptor_ are the energy calculations done in Prime MM-GBSA of the optimized complex (complex), optimized free ligand (ligand), and optimized free receptor (receptor). The OPLS3 force field and VSGB solvation model were used in the calculations. Frames were extracted at every 10 ns of the MD simulations to calculate ‘average dG Bind’.

## 3. Results and discussion

### 3.1. Comparison of RIPK1 and PERK active sites

**Fig. 1:**
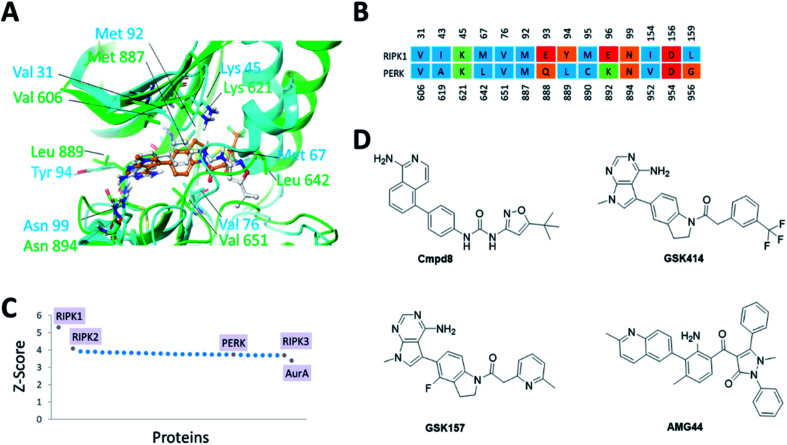
(A) Superposition of binding sites of RIPK1 (PDB ID 4NEU) and PERK (PDB ID 4G31) shown in cyan and green, respectively. (B). Structure-based sequence alignment of the active sites, the sequence numbering (above/below) is based on the PDB structures 4NEU and 4G31 for RIPK1 and PERK, respectively. Residues are colored by residue type, blue is hydrophobic, red is acidic, green is basic and others are orange. (C) Binding sites similar to RIPK1 was calculated using ProBiS server.^24^ PDB/Chain ID: 4NEU.A was used as a query. Selected kinase hits are labeled (see Table S1‡ for the complete list). (D) 2D chemical structures of the small molecule inhibitors of RIPK1 (Cmpd8) and PERK (GSK414, GSK157 and AMG44).

The PERK and RIPK1 crystal structure active sites were superposed to investigate the sequence and structure similarity in the ATP binding region. The binding sites align well, with a C-alpha atoms RMSD of 1.9 Å. Residues Val31, Lys45, Val76, Met92, Asn99, Asp156 (RIPK1 numbering) are conserved between the two systems (Fig. 1). The analysis also revealed small but significant differences between the two binding sites. Instead of the hinge β-strand Cys890 residue in PERK, RIPK1 has Met95 that is engaged in hydrogen bonds with the inhibitor, and Glu93, Tyr94, Glu96, Leu159 in RIPK1 corresponds to residues Gln888, Leu889, Lys892, Gly956 in PERK, respectively. The Cα-helix is closer to the ligand binding site in RIPK1 compared to PERK. In RIPK1, Met67 and Glu63 are oriented towards the active site, Glu63 forms a hydrogen bond with the inhibitor, while Glu62 and Lys65 point away from the binding site. Similarly, in PERK the Cα-helix residues Glu638, Val639 and Leu642 are rotated towards the active site and Lys 640 is pointing out. These differences in the Cα-helix leads to the formation of unique lipophilic pockets that can give rise to different inhibitor interaction landscapes between the two proteins (Fig. 1A and B).

Since structurally similar binding sites are more likely to bind similar scaffolds, we used the ProBiS server^24^ to search for binding sites similar to the RIPK1 active site. As expected we found RIPK1 and RIPK2 structures in the database as top hits with high *Z*-scores of 5.31 and 4.07. PERK is also found among the top hits of binding sites similar to RIPK1, with a Z-score of 3.72 as shown in Fig. 1C. Interestingly, RIPK3 had a lower (yet still very high) Z-score value of 3.69 (Fig. 1C and Table S1‡). The physicochemical properties of the PERK and RIPK1 active sites were calculated, showing similar Dscore and SiteScore values (Table 1). Notably, the AMG44 binding site has a larger volume compared to the other two. Taken together, PERK and RIPK1 display similar binding sites, which explains some of the specificity issues observed experimentally. The compounds GSK414, GSK157 and Cmpd8 are similar in shape with accessible surface area of 666, 652, and 649 Å^2^ respectively (Fig. 1D). Furthermore, GSK414 and GSK157 share a common indoline scaffold, GSK414 has a trifluoromethyl phenyl substituent whereas GSK157 has a methylpyridine substituent. Cmpd8 has an aminoisoquinoline scaffold with a *tert*-butyl oxazole substituent. AMG44 is chemically distinct with a (2-methylquinolin-6-yl)benzoyl group and a diphenyl pyrazol substituent, and a significantly larger accessible surface area of 800 Å^2^.

**Table 1:** Calculated pocket properties^27^ of the kinase active site in the RIPK1 and PERK crystal structures

Protein	PDB code	Ligand name/PDB ID	Dscore	SiteScore	Size	Hydrophilic	Hydrophobic	Volume (Å)^3^
PERK	4G31	GSK414/0WH	1.20	1.14	267	0.71	2.21	521.70
	4M7I	GSK157/27D	1.18	1.13	258	0.79	1.98	569.72
	4X7N	AMG44/3Z5	1.13	1.09	308	0.89	1.55	772.09
RIPK1	4NEU	Cmpd8/Q1A	1.23	1.18	200	0.71	2.96	350.55

### 3.2. Receptor modeling and cross docking of PERK inhibitors

**Fig. 2:**
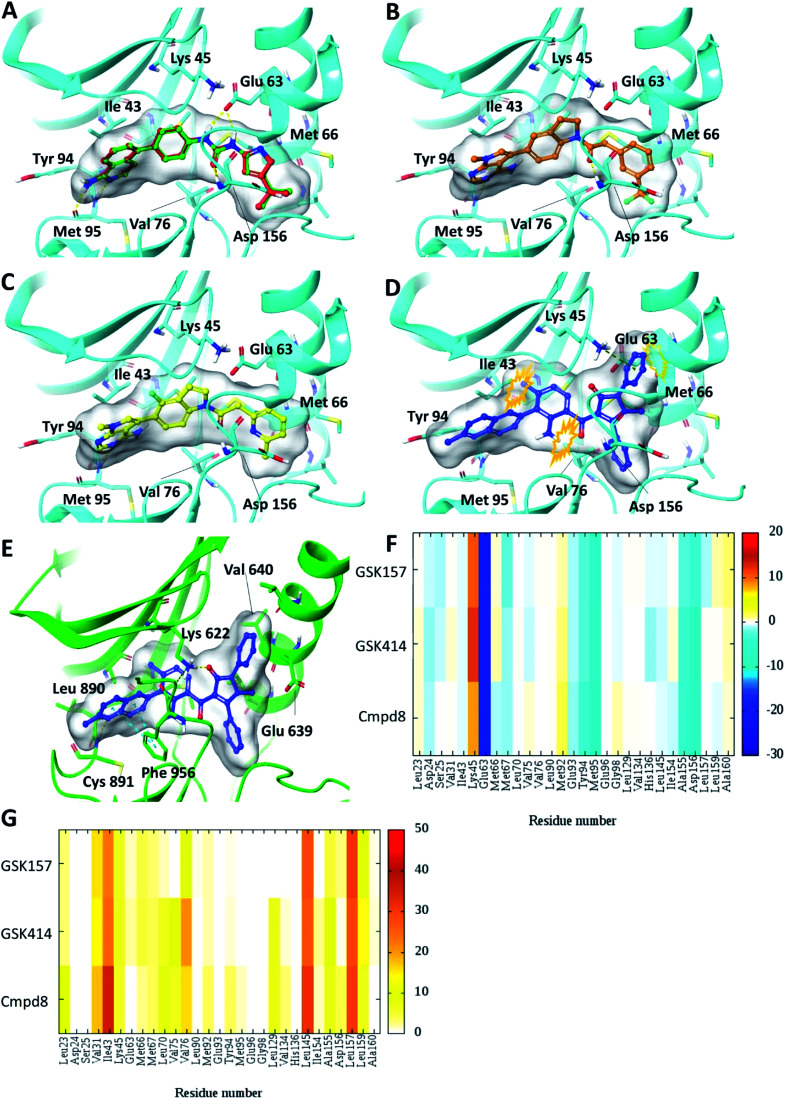
Glide SP Docking. Predicted binding mode of PERK inhibitors GSK414, GSK517 and AMG44 in RIPK1 active site. Close-up view of the RIPK1 active site (PDB ID 4NEU). The protein is rendered with ribbons and key residues are shown as sticks (cyan). (A) Superposition of docked pose (green) of Cmpd8 onto the co-crystallized structure (red). The docked pose of (B) GSK414 (orange), (C) GSK157 (yellow). (D) AMG44 did not dock in the RIPK1. Superposition of the co-crystallized pose of AMG44 in PERK into the RIPK1 active site (blue) shows steric clashes (orange). (E) Co-crystal structure of PERK (green) in complex with AMG44 (blue); PDB ID 4X7N. Per amino-acid interaction energy map of PERK inhibitors docked inside the binding site of RIPK1 using conventional docking studies (PDB ID 4NEU): (F) electrostatic energy values (kcal mol^−1^) and (G) hydrophobic score (arbitrary units).

Self-docking of Cmpd8 in RIPK1 gave a high docking score of −14.8 kcal mol^−1^ and low RMSD of 0.25 Å compared to the crystallographic ligand pose (Fig. 2A) verifying the selected docking methodology. In agreement with the experimental results,^11^ both GSK414 and GSK157 ligands were found to dock to the RIPK1 binding site with strikingly similar poses to its cognate ligand (Cmpd8), and with high docking scores of −13.3 and −11.6 kcal mol^−1^, respectively (Fig. 2B and C). The 3D docking pose of the best conformer for each compound is shown in Fig. 2. In contrast, with conventional molecular docking settings AMG44 did not dock to RIPK1 active site. Superposition of the crystallographic pose of AMG44 bound to PERK into the RIPK1 active site, revealed several clashes which could be the reason for lack of docking (Fig. 2D). Notably, the RIPK1 crystal structure used for docking (PDB 4NEU) is co-crystalized with a relatively small-sized ligand (Cmpd8) and thus the binding pocket might not readily accommodate a bigger ligand in rigid docking. The result obtained is, however, in agreement with the experimental finding that AMG44 has no measurable affinity for RIPK1.^11^

The KIP values were calculated for the docked complexes and the per residue electrostatic and hydrophobic energy contributions depicted as heat maps (Fig. 2F and G). The electrostatic KIPs of the docked structures display several favorable interactions (colored blue) such as Glu63, Glu93, Tyr94, Met95, Ala155, Asp156 common between the GSK compounds and Cmpd8 (Fig. 2F). The compounds also share several hydrophobic interactions (Fig. 2G) including residues such as Val31, Ile43, Leu145, Leu157 (colored brown). The repulsive interaction with Lys45 is observed in all the docked complexes (Fig. 2F). We performed similar analysis and calculated KIP values for the PERK co-crystal structures. The corresponding residue in PERK, Lys621 also showed repulsive interactions with GSK414 and GSK157, whereas it established a favorable electrostatic interaction with AMG44 (Fig. S1‡). To summarize, the KIPs highlight that the PERK inhibitors GSK414 and GSK157 share a binding mode highly similar to the co-crystallized RIPK1 inhibitor.

### 3.3. Induced-fit docking analysis: modeling RIPK1 receptor flexibility produce unique binding mode for AMG44

**Fig. 3:**
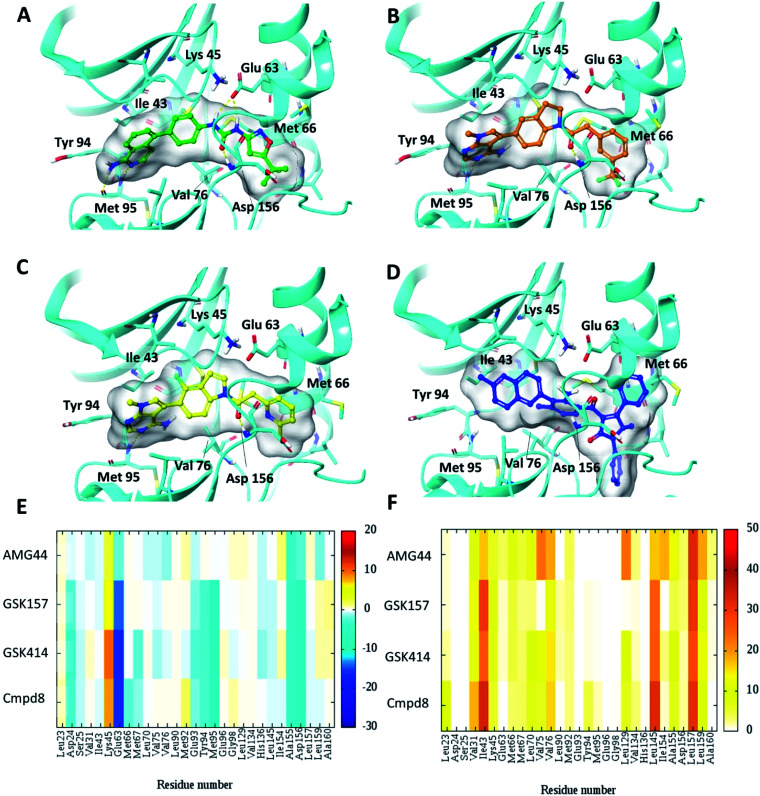
Induced fit docking (IFD) of PERK inhibitors GSK414, GSK517 and AMG44 in the RIPK1 active site. Close-up view of the RIPK1 active site (PDB ID 4NEU). The protein is rendered with ribbons and key residues are shown as sticks (cyan). IFD binding modes of (A) Cmpd8 (green), (B) GSK414 (orange), (C) GSK157 (yellow) show similar binding poses as those obtained using Glide SP. (D) AMG44 (blue) displayed a distinct binding mode compared to the PERK co-crystal (Fig. 2E). Per amino-acid interaction energy map of PERK inhibitors in the binding site of RIPK1 (PDB ID 4NEU) obtained from the IFD docking: (E) electrostatic energy values (kcal mol^−1^) and (F) hydrophobic score (arbitrary units).

To test the impact of receptor relaxation, we performed IFD of the compounds into the RIPK1 binding site. A quantitative analysis of the ligand interactions was made to compare with the results from the Glide binding modes. The GSK414 and GSK157 interaction network of the IFD docked poses were similar to those from the Glide SP docking study (Fig. 3). Interestingly, with the induced-fit docking protocol, AMG44 was able to be accommodated in the RIPK1 active site as the changes in the binding site allowed the diphenylpyrazolidin-3-one ring to be incorporated. However, the compound did not form the canonical H-bond with Met95 despite the high Glide score of −15.5 kcal mol^−1^ and IFD score of −602.9 kcal mol^−1^. AMG44 also lacked the important electrostatic interactions corresponding to residues Asp24, Glu93, Tyr94, Met95 (Fig. 3E and F). In contrast to the favorable Lys621 interaction in the PERK co-crystal (Fig. S1‡), AMG44 induced fit docking in RIPK1 shows a repulsive interaction with Lys45. Collectively, using the active site flexibility, AMG44 is able to attain a docking pose in RIPK1, but with very different interaction pattern compared to Cmpd8, GSK414 and GSK157.

### 3.4. MD simulations analysis

**Fig. 4:**
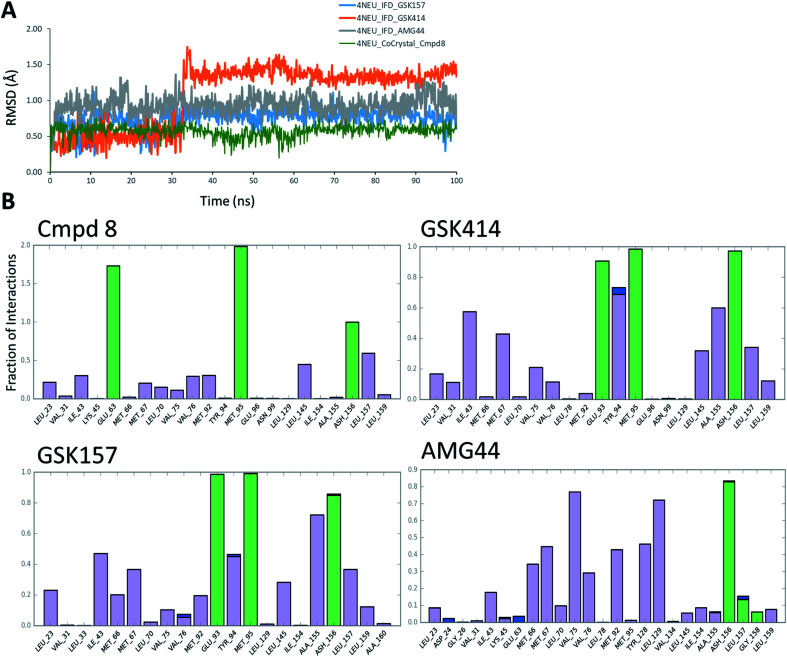
RIPK1 MD simulation analysis. (A) RMSD profiles of the compounds during the MD simulations. (B) Interaction fraction plots depicting protein–ligand contacts during the course of 100 ns MD simulation. H-bonds, hydrophobic interactions and water bridges are shown in green, purple and blue, respectively.

The co-crystal structure of RIPK1 in complex with Cmpd8 and IFD docked poses of GSK414, GSK157 and AMG44 were individually prepared and subjected to MD simulations using the Desmond program.^34^ MD simulations were performed for a total of 100 ns and trajectories were analyzed for ligand RMSD values and interaction fingerprints of the compounds in the active site over time as shown in Fig. 4.

The native RIPK1 ligand Cmpd8 isoquinolin-1-amine ring establishes a strong pair of hydrogen bonds with Met95 throughout the simulation. Additional hydrogen bonds with Glu63 and Asp156 are constantly present and contribute to the overall ligand stability. Other prominent ligand contacts in the binding site include Val76, Met92, Leu145, Leu157. The same interaction trend was observed in the GSK414 simulation where the pyrimidin-4-amine ring engages in hydrogen bonds with Met95 and Glu93. The hydrogen bond with Asp156 is also consistent during the simulation, as were the hydrophobic contacts with Leu145, Leu157. The key difference compared with the Cmpd8 is the absence of the Glu63 hydrogen bond. Additional GSK414 contacts include Ile43, Met67, Tyr94 and Ala155. Similarly, GSK157 also engages in hydrogen bonds with Met95, Glu93 and Asp156. The hydrophobic interactions include Met67, Tyr94, Leu145, Ala155 and Leu157. The strong and consistent interaction patterns observed with GSK414, GKS157 and the low RMSD deviations support the tight-binding nature of the ligands in the RIPK1 active site. In general, the hydrogen bonding was largely mediated by Glu63, Glu93, Met95 and Asp156 residues in the active site.

In contrast, the same interaction pattern was not observed in the AMG44 simulation. The compound did not engage in a hydrogen bond with Met95 or Glu93 (Fig. 4B). Also there were no hydrophobic interactions with the residues Leu145, Ala155 and Leu157. Instead, the ligand was stabilized through alternative hydrophobic interactions with Met66, Met67, Val75, Met92, Tyr128 and Leu129 caused by the structural changes during the IFD procedure. The intermolecular hydrogen bonds determined for each frame of the 100 ns MD simulation are depicted in Fig. S3.‡ The number of hydrogen bonds formed by Cmpd8, GSK414 and GSK157 systems are larger than the AMG44 system (Fig. S3‡) with an average of 4.71, 2.86, 2.82, and 0.96 respectively. Despite acquiring a strikingly different interaction profile, AMG44 was stable during the simulation with a low ligand RMSD (Fig. 4A).

In addition, active site geometric stability analysis (RMSD) was performed on the simulated trajectories in order to compare the active site fluctuations upon ligand binding. AMG44 showed a higher RMSD with an average of 2.86 Å compared to the other compounds as shown in Fig. S2.‡ The hydrogen bond analysis and active site geometric stability analysis indicate that Cmpd8, GSK414, and GSK157 are more stable during the MD simulation compared to AMG44.

MMGBSA binding free energies were calculated for the single best docked structure and the ensemble-average structures from the MD simulation. The results along with the corresponding docking scores are listed in Table S2.‡ In general, the calculated binding energies are in the same range for all the compounds. Notably, compared to the other compounds, AMG44 has a lower Coulomb energy component (MMGBSA dG Bind Coulomb); −5.86 kcal mol^−1^ for single IFD docked structure and −10.91 kcal mol^−1^ in MD based calculation (Table S2‡). The weak electrostatic interaction of AMG44 (as shown in Fig. 3E) from the induced fit docking correlates with the low Coulomb energy obtained from the MMGBSA calculations. Taken together, these data show that GSK414 and GSK157 have similar interaction profiles as the RIPK1 native ligand, whereas AMG44 binds differently in the binding site of RIPK1.

## 4. Conclusions and perspective

Selectivity is a major concern in kinase drug discovery. The concept of polypharmacology associated with kinase inhibitors has gained much interest in cancer therapy.^35–37^ The clinical efficacy of kinase inhibitors however does not solely depend on their selectivity profiles, but is also dependent on other factors such as potency, tissue distribution, toxicity, and drug resistance. A less selective inhibitor may in some cases be therapeutically beneficial based on functional activity *versus* off-target effects and their biological relevance. To this end, off-target effects must be carefully evaluated in order to broaden the clinical applications of inhibitors and avoid unwanted clinical outcomes.

In this study, using a computational molecular modeling approach, we have determined the potential binding modes of PERK inhibitors GSK414 and GSK157 in the RIPK1 active site. PERK and RIPK1 kinase binding sites showed high structure and sequence similarities. Selectivity to either target can be achieved by accounting for the differences in the electrostatic potential interaction profiles in the binding site. Consistent with the available experimental studies, molecular docking was able to correctly predict the dual targeting nature of GSK414 and GSK157 under conventional molecular docking and IFD settings. IFD of AMG44 resulted in a unique binding mode with a good docking score and MMGBSA binding energy, but that lacked the canonical interaction in the active site and displayed higher active site flexibility in the MD simulation. While the computational analysis alone cannot rule out the possibility of the AMG44 binding to RIPK1, we speculate that the weak electrostatic interactions is a main reason for lack of RIPK1 inhibition by AMG44.^11^ Weak electrostatic interactions can limit a compound's ability to compete with the endogenous ATP, resulting in no functional effect in kinase assays.

Despite not engaging in any crucial ligand interactions, the AMG44 still had a high docking score highlighting the limitations of the scoring functions.^38,39^ Therefore, counter screens using docking methods should be carefully evaluated and the results should not be prioritized based on docking scores alone. In particular, the results show possibilities for further improvement of RIPK1 inhibitors by investigating substitutions patterns that could mediate favorable interaction with Lys45. A consensus scoring may help reduce the number of false positives predictions,^38,40^ and prior knowledge of ligand interactions and custom scoring functions can improve the docking accuracy.^41^ In addition, long MD timescales are often needed to rule out false binding.^42^ The strategies used in the current study provide a computational toolkit to assess the similarity of protein binding sites and thereby predict the potential off-target interactions of small molecule inhibitors.

## Author contributions

All authors conceived the study. C. C. and A. C. performed the calculations and wrote the first draft. All authors finalized the submitted manuscript.

## Conflicts of interest

A. M. G., A. S. and L. A. E. are co-founders of Cell Stress Discoveries, Ltd. The authors declare no conflicting interests.

## Supplementary Material

RA-010-C9RA08047C-s001
