# Supplementary material for: Molecular modeling provides a structural basis for PERK inhibitor selectivity towards RIPK1
Source: RSC Adv. 2020 Jan 2;10(1):367–75. doi: 10.1039/c9ra08047c (PMC9092956; doi:10.1039/c9ra08047c)
Supplement: RA-010-C9RA08047C-s001 [file RA-010-C9RA08047C-s001.pdf]

Electronic Supplementary Material (ESI) for RSC Advances.
This journal is © The Royal Society of Chemistry 2019

Molecular modeling provides structural basis for PERK inhibitor selectivity towards RIPK1

Chetan Chintha[1#], Antonio Carlesso[2#], Adrienne M. Gorman[1], Afshin Samali[1], Leif A. Eriksson[2*]

[1]Apoptosis Research Centre, National University of Ireland Galway, Galway, Ireland.
[2]Department of Chemistry and Molecular Biology, University of Gothenburg, 405 30 Göteborg, Sweden

#These authors contributed equally to this work.

[*]*Correspondence*
Leif Eriksson, Department of Chemistry and Molecular Biology,
University of Gothenburg, 405 30 Göteborg, Sweden.
Email: leif.eriksson@chem.gu.se

SUPPLEMENTARY INFORMATION

Page

Table S1. List of proteins with binding site similar to RIPK1 as calculated by the ProBiS sever[1] **S2-S11**

Table S2: Calculated docking and binding affinity (MMGBSA) scores of compounds for RIPK1 (PDB 4NEU) active site using different strategies. (A) The Glide SP docked poses. (B) Induced fit docking (IFD) poses. (C) Protein-ligand frames at every 10ns from the 100ns MD simulation was used for MMGBSA calculation, SD stands for standard deviation and N/A for not applicable, where no valid poses were obtained. **S12**

Figure S1. Per amino-acid interaction energy map for PERK co-crystal structures: crystal structure of GSK6414 Bound (PDB code: 4G31), crystal structure of GSK6157 bound (PDB code: 4M7I) and co-crystal structure of PERK bound to AMG44 (PDB code: 4X7N). (A) Electrostatic energy values (kcal/mol). (B) Hydrophobic score (arbitrary units). **S13**

Figure S2. Active site dynamics of the RIPK1 complexes during 100ns MD simulations. RMSD of the active site residues is plotted as a function of MD simulation time. **S13**

Figure S3. Comparison of the number of hydrogen bonds (H-bonds) formed by the compounds at the RIPK1 active site during the 100 ns MD simulation. **S14**

Bibliography **S14**

Table S1. List of proteins with binding site similar to RIPK1 as calculated by the ProBiS sever[1]

PDB	Protein Name	UniProt ID	Z-Score
4neu	RECEPTOR-INTERACTING SERINE/THREONINE-PROTEIN KINASE 1	Q13546	5.3
4itj	RECEPTOR-INTERACTING SERINE/THREONINE-PROTEIN KINASE 1	Q13546	4.7
4c8b	RECEPTOR-INTERACTING SERINE/THREONINE-PROTEIN KINASE 2	O43353	4.1
3dtc	MITOGEN-ACTIVATED PROTEIN KINASE KINASE KINASE 9	P80192	3.9
3soc	ACTIVIN RECEPTOR TYPE-2A	P27037	3.9
3u4w	PROTO-ONCOGENE TYROSINE-PROTEIN KINASE SRC	P00523	3.9
4csv	SRC-ABL TYROSINE KINASE ANCESTOR	N/A	3.9
4x2j	TGF-BETA RECEPTOR TYPE-1	P36897	3.9
4hcu	TYROSINE-PROTEIN KINASE ITK/TSK	Q08881	3.9
2eva	TAK1 KINASE - TAB1 CHIMERA FUSION PROTEIN	O43318	3.8
5c9c	SERINE/THREONINE-PROTEIN KINASE B-RAF	P15056	3.8
1qpc	LCK KINASE	P06239	3.8
4kik	INHIBITOR OF NUCLEAR FACTOR KAPPA-B KINASE SUBUNIT BETA	O14920	3.8
3pls	MACROPHAGE-STIMULATING PROTEIN RECEPTOR	Q04912	3.8
1byg	PROTEIN (C-TERMINAL SRC KINASE)	P41240	3.8
3zfx	EPHRIN TYPE-B RECEPTOR 1	P54762	3.8
2xik	SERINE/THREONINE PROTEIN KINASE 25	O00506	3.8
4cqe	SLC45A3-BRAF FUSION PROTEIN	D7PBN4	3.8
3q4u	ACTIVIN RECEPTOR TYPE-1	Q04771	3.8
1p4o	INSULIN-LIKE GROWTH FACTOR I RECEPTOR PROTEIN	P08069	3.8
3hng	VASCULAR ENDOTHELIAL GROWTH FACTOR RECEPTOR 1	P17948	3.8
4y5q	CALMODULIN-DOMAIN PROTEIN KINASE 1, PUTATIVE	A3FQ16	3.7
4pdp	SERINE/THREONINE-PROTEIN KINASE RAD53	P22216	3.7
4xi2	TYROSINE-PROTEIN KINASE BTK	P35991	3.7
4x7k	EUKARYOTIC TRANSLATION INITIATION FACTOR 2-ALPHA KINASE 3	Q9NZJ5	3.7
4yzn	PROBABLE SERINE/THREONINE-PROTEIN KINASE ROCO4	Q6XHB2	3.7
4ymj	NT-3 GROWTH FACTOR RECEPTOR	Q16288	3.7
3lzb	EPIDERMAL GROWTH FACTOR RECEPTOR	P00533	3.7
4clj	ALK TYROSINE KINASE RECEPTOR	Q9UM73	3.7
3zbf	PROTO-ONCOGENE TYROSINE-PROTEIN KINASE ROS	P08922	3.7
2psq	FIBROBLAST GROWTH FACTOR RECEPTOR 2	P21802	3.7
4m66	RECEPTOR-INTERACTING SERINE/THREONINE-PROTEIN KINASE 3	Q9QZL0	3.7
3vhe	VASCULAR ENDOTHELIAL GROWTH FACTOR RECEPTOR 2	P35968	3.7
3p86	SERINE/THREONINE-PROTEIN KINASE CTR1	Q05609	3.7
2r0i	SERINE/THREONINE-PROTEIN KINASE MARK2	O08679	3.7
1jpa	NEURAL KINASE, NUK=EPH/ELK/ECK FAMILY RECEPTOR-LIKE TYROSINE KINASE	P54763	3.7

2vd5	DMPK PROTEIN	Q09013	3.7
1t46	HOMO SAPIENS V-KIT HARDY-ZUCKERMAN 4 FELINE SARCOMA VIRAL ONCOGENE HOMOLOG	P10721	3.7
3vo3	VASCULAR ENDOTHELIAL GROWTH FACTOR RECEPTOR 2	P35968	3.7
3is5	CALCIUM-DEPENDENT PROTEIN KINASE	N/A	3.7
4qqt	FIBROBLAST GROWTH FACTOR RECEPTOR 4	P22455	3.7
2hk5	TYROSINE-PROTEIN KINASE HCK	P08631	3.7
2qkw	PROTEIN KINASE	Q40234	3.7
3hmi	TYROSINE-PROTEIN KINASE ABL2	P42684	3.7
3s95	LIM DOMAIN KINASE 1	P53667	3.7
1blx	CYCLIN-DEPENDENT KINASE 6	Q00534	3.7
4ase	VASCULAR ENDOTHELIAL GROWTH FACTOR RECEPTOR 2	P35968	3.7
1opk	PROTO-ONCOGENE TYROSINE-PROTEIN KINASE ABL1	P00520	3.7
4aaa	CYCLIN-DEPENDENT KINASE-LIKE 2	Q92772	3.7
3lxp	NON-RECEPTOR TYROSINE-PROTEIN KINASE TYK2	P29597	3.7
2z7r	RIBOSOMAL PROTEIN S6 KINASE ALPHA-1	Q15418	3.6
2h8h	PROTO-ONCOGENE TYROSINE-PROTEIN KINASE SRC	P12931	3.6
1phk	PHOSPHORYLASE KINASE	P00518	3.6
4rt7	RECEPTOR-TYPE TYROSINE-PROTEIN KINASE FLT3	P36888	3.6
3omv	RAF PROTO-ONCOGENE SERINE/THREONINE-PROTEIN KINASE	P04049	3.6
1k9a	CARBOXYL-TERMINAL SRC KINASE	P32577	3.6
1rjb	FL CYTOKINE RECEPTOR	P36888	3.6
4pl3	SERINE/THREONINE-PROTEIN KINASE/ENDORIBONUCLEASE IRE1	Q9EQY0	3.6
4gt4	TYROSINE-PROTEIN KINASE TRANSMEMBRANE RECEPTOR ROR2	Q01974	3.6
4y93	NON-SPECIFIC PROTEIN-TYROSINE KINASE,NON-SPECIFIC PROTEIN-TYROSINE KINASE	Q3ZC95	3.6
1fot	CAMP-DEPENDENT PROTEIN KINASE TYPE 1	P06244	3.6
1qcf	HAEMATOPOETIC CELL KINASE (HCK)	P08631	3.6
3lxl	TYROSINE-PROTEIN KINASE JAK3	P52333	3.6
4usf	STE20-LIKE SERINE/THREONINE-PROTEIN KINASE	Q9H2G2	3.6
2i1m	MACROPHAGE COLONY-STIMULATING FACTOR 1 RECEPTOR	P07333	3.6
4oli	NON-RECEPTOR TYROSINE-PROTEIN KINASE TYK2	P29597	3.6
2zv2	CALCIUM/CALMODULIN-DEPENDENT PROTEIN KINASE KINASE 2	Q96RR4	3.6
4crs	SERINE/THREONINE-PROTEIN KINASE N2	Q16513	3.6
4at5	BDNF/NT-3 GROWTH FACTORS RECEPTOR	Q16620	3.6
3dpk	MACROPHAGE COLONY-STIMULATING FACTOR 1 RECEPTOR	P11362	3.6
3bu3	INSULIN RECEPTOR SUBUNIT BETA	P06213	3.5
4fg8	CALCIUM/CALMODULIN-DEPENDENT PROTEIN KINASE TYPE 1	Q14012	3.5
3ugc	TYROSINE-PROTEIN KINASE JAK2	O60674	3.5
1o6l	RAC-BETA SERINE/THREONINE PROTEIN KINASE	P31751	3.5
4i4e	FOCAL ADHESION KINASE 1	Q05397	3.5
4rfz	TYROSINE-PROTEIN KINASE BTK	Q06187	3.5
4eqm	PROTEIN KINASE	A0A0H3JME9	3.5

4pmp	HIGH AFFINITY NERVE GROWTH FACTOR RECEPTOR	P04629	3.5
2wtk	SERINE/THREONINE-PROTEIN KINASE 11	Q15831	3.5
2vwx	EPHRIN TYPE-B RECEPTOR 4	P54760	3.5
3b2t	FIBROBLAST GROWTH FACTOR RECEPTOR 2	P21802	3.5
2i6l	MITOGEN-ACTIVATED PROTEIN KINASE 6	Q16659	3.5
3kfa	TYROSINE-PROTEIN KINASE ABL1	P00520	3.5
3zdu	CYCLIN-DEPENDENT KINASE-LIKE 3	Q8IVW4	3.5
4e1z	NON-RECEPTOR TYROSINE-PROTEIN KINASE TYK2	Q9R117	3.5
2r2p	EPHRIN TYPE-A RECEPTOR 5	P54756	3.5
4xuf	RECEPTOR-TYPE TYROSINE-PROTEIN KINASE FLT3	P36888	3.5
3pfq	PROTEIN KINASE C BETA TYPE	P68403	3.5
2rei	EPHRIN TYPE-A RECEPTOR 7	Q15375	3.5
2w4o	CALCIUM/CALMODULIN-DEPENDENT PROTEIN KINASE TYPE IV	Q16566	3.5
4czu	CBL-INTERACTING SERINE/THREONINE-PROTEIN KINASE 23	Q93VD3	3.5
2y6o	EPHRIN TYPE-A RECEPTOR 4	Q03137	3.5
3mdy	BONE MORPHOGENETIC PROTEIN RECEPTOR TYPE-1B	O00238	3.5
4k33	FIBROBLAST GROWTH FACTOR RECEPTOR 3	P22607	3.5
3rp9	MITOGEN-ACTIVATED PROTEIN KINASE	B6KP12	3.5
3fme	DUAL SPECIFICITY MITOGEN-ACTIVATED PROTEIN KINASE KINASE 6	P52564	3.5
4ct2	3-PHOSPHOINOSITIDE-DEPENDENT PROTEIN KINASE 1	O15530	3.5
3g2f	BONE MORPHOGENETIC PROTEIN RECEPTOR TYPE-2	Q13873	3.5
3my0	SERINE/THREONINE-PROTEIN KINASE RECEPTOR R3	P37023	3.5
3i6u	SERINE/THREONINE-PROTEIN KINASE CHK2	O96017	3.5
4yhj	G PROTEIN-COUPLED RECEPTOR KINASE 4	P32298	3.5
4lg4	SERINE/THREONINE-PROTEIN KINASE 3	Q13188	3.5
3uiu	INTERFERON-INDUCED, DOUBLE-STRANDED RNA-ACTIVATED PROTEIN KINASE	P19525	3.4
4wun	FIBROBLAST GROWTH FACTOR RECEPTOR 1	P11362	3.4
2dq7	PROTO-ONCOGENE TYROSINE-PROTEIN KINASE FYN	P06241	3.4
3kmu	INTEGRIN-LINKED KINASE	Q13418	3.4
3tt0	BASIC FIBROBLAST GROWTH FACTOR RECEPTOR 1	P11362	3.4
4tnd	G PROTEIN-COUPLED RECEPTOR KINASE 5	P34947	3.4
4red	5'-AMP-ACTIVATED PROTEIN KINASE CATALYTIC SUBUNIT ALPHA-1	Q13131	3.4
3brb	PROTO-ONCOGENE TYROSINE-PROTEIN KINASE MER	Q12866	3.4
2bdw	HYPOTHETICAL PROTEIN K11E8.1D	O62305	3.4
2qol	EPHRIN RECEPTOR	P29320	3.4
4jdi	SERINE/THREONINE-PROTEIN KINASE PAK 4	O96013	3.4
4y0x	SERINE/THREONINE-PROTEIN KINASE PKNG	P9WI73	3.4
4w9w	BMP-2-INDUCIBLE PROTEIN KINASE	Q9NSY1	3.4
4aw2	SERINE/THREONINE-PROTEIN KINASE MRCK ALPHA	O54874	3.4
4ejn	RAC-ALPHA SERINE/THREONINE-PROTEIN KINASE	P31749	3.4
4wb7	DNAJ HOMOLOG SUBFAMILY B MEMBER 1,CAMP-DEPENDENT PROTEIN KINASE CATALYTIC SUBUNIT ALPHA	P25685	3.4

3ork	SERINE/THREONINE PROTEIN KINASE	A5TY84	3.4
4fyo	TYROSINE-PROTEIN KINASE SYK	P43405	3.4
4xey	TYROSINE-PROTEIN KINASE ABL1	P00519	3.4
1mqb	EPHRIN TYPE-A RECEPTOR 2	P29317	3.4
2j0j	FOCAL ADHESION KINASE 1	Q00944	3.4
3uto	TWITCHIN	Q23551	3.4
3dxn	CALMODULIN-LIKE DOMAIN PROTEIN KINASE ISOFORM 3	Q3HNM6	3.4
2h6d	5'-AMP-ACTIVATED PROTEIN KINASE CATALYTIC SUBUNIT ALPHA-2	P54646	3.4
2jc6	CALCIUM/CALMODULIN-DEPENDENT PROTEIN KINASE TYPE 1D	Q8IU85	3.4
4j8m	AURORA KINASE A	O14965	3.4
3sxs	CYTOPLASMIC TYROSINE-PROTEIN KINASE BMX	P51813	3.4
3fe3	MAP/MICROTUBULE AFFINITY-REGULATING KINASE 3	P27448	3.4
2gcd	SERINE/THREONINE-PROTEIN KINASE TAO2	Q9JLS3	3.4
1u5r	SERINE/THREONINE PROTEIN KINASE TAO2	Q9JLS3	3.4
3ggf	SERINE/THREONINE-PROTEIN KINASE MST4	Q9P289	3.4
4bkj	EPITHELIAL DISCOIDIN DOMAIN-CONTAINING RECEPTOR 1	Q08345	3.4
3lij	CALCIUM/CALMODULIN DEPENDENT PROTEIN KINASE WITH A KINASE DOMAIN AND 4 CALMODULIN LIKE EF HANDS	Q5CS01	3.4
3q5i	PROTEIN KINASE	Q4YRR5	3.4
3c4z	RHODOPSIN KINASE	P28327	3.4
3vn9	DUAL SPECIFICITY MITOGEN-ACTIVATED PROTEIN KINASE KINASE 6	P52564	3.4
4x3f	SERINE/THREONINE-PROTEIN KINASE PKNA	P9WI83	3.4
2oib	INTERLEUKIN-1 RECEPTOR-ASSOCIATED KINASE 4	Q9NWZ3	3.4
4js8	DUAL SPECIFICITY PROTEIN KINASE TTK	P33981	3.4
4wih	CAMP-DEPENDENT PROTEIN KINASE CATALYTIC SUBUNIT ALPHA	P25321	3.4
2a19	INTERFERON-INDUCED, DOUBLE-STRANDED RNA-ACTIVATED PROTEIN KINASE	P19525	3.4
2zmd	DUAL SPECIFICITY PROTEIN KINASE TTK	P33981	3.4
2yex	SERINE/THREONINE-PROTEIN KINASE CHK1	O14757	3.4
4yc6	CYCLIN-DEPENDENT KINASE 1	P06493	3.3
4idt	MITOGEN-ACTIVATED PROTEIN KINASE KINASE KINASE 14	Q99558	3.3
1koa	TWITCHIN	Q23551	3.3
4rgj	CALCIUM-DEPENDENT PROTEIN KINASE 4	Q8IBS5	3.3
4bfm	MATERNAL EMBRYONIC LEUCINE ZIPPER KINASE	Q61846	3.3
4ci6	PROTEIN KINASE YOPO	Q93KQ6	3.3
3uc3	SERINE/THREONINE-PROTEIN KINASE SRK2I	Q39193	3.3
1ob3	CELL DIVISION CONTROL PROTEIN 2 HOMOLOG	Q07785	3.3
4c2v	AURORA KINASE B-A	Q6DE08	3.3
2wqm	SERINE/THREONINE-PROTEIN KINASE NEK7	Q8TDX7	3.3
3a99	PROTO-ONCOGENE SERINE/THREONINE-PROTEIN KINASE PIM-1	P11309	3.3
2fst	MITOGEN-ACTIVATED PROTEIN KINASE 14	Q16539	3.3
3hmp	DUAL SPECIFICITY PROTEIN KINASE TTK	P33981	3.3
4rpv	SERINE/THREONINE-PROTEIN KINASE PIM-1	P11309	3.3

3gp0	MITOGEN-ACTIVATED PROTEIN KINASE 11	Q15759	3.3
3oz6	MITOGEN-ACTIVATED PROTEIN KINASE 1, SERINE/THREONINE PROTEIN KINASE	A3FQ79	3.3
2acx	G PROTEIN-COUPLED RECEPTOR KINASE 6	P43250	3.3
2zv7	TYROSINE-PROTEIN KINASE LYN	P25911	3.3
3lcd	MACROPHAGE COLONY-STIMULATING FACTOR 1 RECEPTOR	P07333	3.3
4krd	CYCLIN-DEPENDENT PROTEIN KINASE PHO85	P17157	3.3
4i5p	SERINE/THREONINE-PROTEIN KINASE PLK2	Q9NYY3	3.3
4fl3	TYROSINE-PROTEIN KINASE SYK	P43405	3.3
3qup	TYROSINE-PROTEIN KINASE RECEPTOR TYRO3	P55144	3.3
1fmk	TYROSINE-PROTEIN KINASE SRC	P12931	3.3
3com	SERINE/THREONINE-PROTEIN KINASE 4	Q13043	3.3
4ow8	SERINE/THREONINE-PROTEIN KINASE PKNA	P9WI82	3.3
3hko	CALCIUM/CALMODULIN-DEPENDENT PROTEIN KINASE WITH A KINASE DOMAIN AND 2 CALMODULIN-LIKE EF HANDS	Q5CZ29	3.3
2f2u	RHO-ASSOCIATED PROTEIN KINASE 2	Q28021	3.3
4tpt	LIM DOMAIN KINASE 2	P53671	3.3
4bky	MATERNAL EMBRYONIC LEUCINE ZIPPER KINASE	Q14680	3.3
1xjd	PROTEIN KINASE C, THETA TYPE	Q04759	3.3
4rer	5'-AMP-ACTIVATED PROTEIN KINASE CATALYTIC SUBUNIT ALPHA-1	Q13131	3.3
3v8s	RHO-ASSOCIATED PROTEIN KINASE 1	Q13464	3.3
3lm5	SERINE/THREONINE-PROTEIN KINASE 17B	O94768	3.2
3mtl	CELL DIVISION PROTEIN KINASE 16	Q00536	3.2
4yno	MITOGEN-ACTIVATED PROTEIN KINASE 13	O15264	3.2
3eb0	PUTATIVE UNCHARACTERIZED PROTEIN	A3FQN0	3.2
4f6u	CYCLIN-DEPENDENT KINASE 8	P49336	3.2
2pzi	PROBABLE SERINE/THREONINE-PROTEIN KINASE PKNG	P9WI73	3.2
4d28	CBL-INTERACTING SERINE/THREONINE-PROTEIN KINASE 24	Q9LDI3	3.2
2i0e	PROTEIN KINASE C-BETA II	P05771	3.2
1zy4	SERINE/THREONINE-PROTEIN KINASE GCN2	P15442	3.2
2v7o	CALCIUM/CALMODULIN-DEPENDENT PROTEIN KINASE TYPE II GAMMA CHAIN	Q13555	3.2
4qmt	SERINE/THREONINE-PROTEIN KINASE 24	Q9Y6E0	3.2
3hzt	CALCIUM-DEPENDENT PROTEIN KINASE 3	Q3HNM6	3.2
3rzf	MGC80376 PROTEIN	Q6INT1	3.2
1cm8	PHOSPHORYLATED MAP KINASE P38-GAMMA	P53778	3.2
4r1v	HEPATOCYTE GROWTH FACTOR RECEPTOR	P08581	3.2
3uim	BRASSINOSTEROID INSENSITIVE 1-ASSOCIATED RECEPTOR KINASE 1	Q94F62	3.2
3dls	PAS DOMAIN-CONTAINING SERINE/THREONINE-PROTEIN KINASE	Q96RG2	3.2
2ac3	MAP KINASE-INTERACTING SERINE/THREONINE KINASE 2	Q9HBH9	3.2
4obo	MITOGEN-ACTIVATED PROTEIN KINASE KINASE KINASE KINASE 4	O95819	3.2
3dak	SERINE/THREONINE-PROTEIN KINASE OSR1	O95747	3.2
2qlu	ACTIVIN RECEPTOR TYPE IIB	Q13705	3.2

3v5w	G-PROTEIN COUPLED RECEPTOR KINASE 2	P25098	3.2
4wsq	AP2-ASSOCIATED PROTEIN KINASE 1	Q2M2I8	3.2
4mvf	CALCIUM-DEPENDENT PROTEIN KINASE 2	O15865	3.2
3cek	DUAL SPECIFICITY PROTEIN KINASE TTK	P33981	3.2
2y7j	PHOSPHORYLASE B KINASE GAMMA CATALYTIC CHAIN, TESTIS/LIVER ISOFORM	P15735	3.2
3kul	EPHRIN TYPE-A RECEPTOR 8	P29322	3.2
4p2k	EPHRIN TYPE-A RECEPTOR 2	P29317	3.2
3zfy	EPHRIN TYPE-B RECEPTOR 3	P54753	3.2
1q8y	SR PROTEIN KINASE	Q03656	3.2
2y4i	KINASE SUPPRESSOR OF RAS 2	Q6VAB6	3.2
1luf	MUSCLE-SPECIFIC TYROSINE KINASE RECEPTOR MUSK	Q62838	3.2
4b6l	SERINE/THREONINE-PROTEIN KINASE PLK3	Q9H4B4	3.2
3p1a	MEMBRANE-ASSOCIATED TYROSINE- AND THREONINE-SPECIFIC CDC2-INHIBITORY KINASE	Q99640	3.2
3bkb	PROTO-ONCOGENE TYROSINE-PROTEIN KINASE FES/FPS	P07332	3.2
2j0i	SERINE/THREONINE-PROTEIN KINASE PAK 4	O96013	3.2
3gbz	KINASE, CMGC CDK	A8BZ95	3.2
3wf7	RIBOSOMAL PROTEIN S6 KINASE BETA-1	P23443	3.2
2wei	CALMODULIN-DOMAIN PROTEIN KINASE 1, PUTATIVE	A3FQ16	3.2
3niz	RHODANESE FAMILY PROTEIN	Q5CRJ8	3.2
4oh4	PROTEIN BRASSINOSTEROID INSENSITIVE 1	O22476	3.2
2ycf	SERINE/THREONINE-PROTEIN KINASE CHK2	O96017	3.1
2eu9	DUAL SPECIFICITY PROTEIN KINASE CLK3	P49761	3.1
4btf	MIXED LINEAGE KINASE DOMAIN-LIKE PROTEIN	Q9D2Y4	3.1
3bhy	DEATH-ASSOCIATED PROTEIN KINASE 3	O43293	3.1
3n9x	PHOSPHOTRANSFERASE	Q4Z5A3	3.1
3fpq	SERINE/THREONINE-PROTEIN KINASE WNK1	Q9JIH7	3.1
3ma6	CALMODULIN-DOMAIN PROTEIN KINASE 1	Q9BJF5	3.1
3hyh	CARBON CATABOLITE-DEREPRESSING PROTEIN KINASE	P06782	3.1
4agu	CYCLIN-DEPENDENT KINASE-LIKE 1	Q00532	3.1
2hak	SERINE/THREONINE-PROTEIN KINASE MARK1	Q9P0L2	3.1
3txo	PROTEIN KINASE C ETA TYPE	P24723	3.1
4euu	SERINE/THREONINE-PROTEIN KINASE TBK1	Q9UHD2	3.1
4o38	CYCLIN-G-ASSOCIATED KINASE	O14976	3.1
4m68	MIXED LINEAGE KINASE DOMAIN-LIKE PROTEIN	Q9D2Y4	3.1
3poz	EPIDERMAL GROWTH FACTOR RECEPTOR	P00533	3.1
4qny	MITOGEN ACTIVATED PROTEIN KINASE, PUTATIVE	E9BQ78	3.1
3nr9	DUAL SPECIFICITY PROTEIN KINASE CLK2	P49760	3.1
1u59	TYROSINE-PROTEIN KINASE ZAP-70	P43403	3.1
4oth	SERINE/THREONINE-PROTEIN KINASE N1	Q16512	3.1
2r4b	RECEPTOR TYROSINE-PROTEIN KINASE ERBB-4	Q15303	3.1
2dyl	DUAL SPECIFICITY MITOGEN-ACTIVATED PROTEIN KINASE KINASE 7	O14733	3.1

2h34	SERINE/THREONINE-PROTEIN KINASE PKNE	P9WI77	3.1
3c1x	HEPATOCYTE GROWTH FACTOR RECEPTOR	P08581	3.1
2xrw	MITOGEN-ACTIVATED PROTEIN KINASE 8	P45983	3.1
2x7f	TRAF2 AND NCK-INTERACTING PROTEIN KINASE	Q9UKE5	3.1
4hzs	ACTIVATED CDC42 KINASE 1	Q07912	3.1
4ckj	PROTO-ONCOGENE TYROSINE-PROTEIN KINASE RECEPTOR RET	P07949	3.1
4o2z	MITOGEN-ACTIVATED PROTEIN KINASE 3, PUTATIVE	E9BA99	3.1
2w5a	SERINE/THREONINE-PROTEIN KINASE NEK2	P51955	3.1
4fie	SERINE/THREONINE-PROTEIN KINASE PAK 4	O96013	3.1
4fkl	CYCLIN-DEPENDENT KINASE 2	P24941	3.1
4fr4	SERINE/THREONINE-PROTEIN KINASE 32A	Q8WU08	3.1
3cc6	PROTEIN TYROSINE KINASE 2 BETA	Q14289	3.1
4lgd	SERINE/THREONINE-PROTEIN KINASE 3	Q13188	3.1
4m3a	STRESS-ACTIVATED PROTEIN KINASE JNK	P92208	3.1
4qfr	5'-AMP-ACTIVATED PROTEIN KINASE CATALYTIC SUBUNIT ALPHA-1	P54645	3.1
4x7q	SERINE/THREONINE-PROTEIN KINASE PIM-2	Q9P1W9	3.1
2wel	CALCIUM/CALMODULIN-DEPENDENT PROTEIN KINASE TYPE II DELTA CHAIN	Q13557	3.1
3e7o	MITOGEN-ACTIVATED PROTEIN KINASE 9	P45984	3.1
4b9d	SERINE/THREONINE-PROTEIN KINASE NEK1	Q96PY6	3.0
2r5t	SERINE/THREONINE-PROTEIN KINASE SGK1	O00141	3.0
3udb	SERINE/THREONINE-PROTEIN KINASE SRK2E	Q940H6	3.0
1z57	DUAL SPECIFICITY PROTEIN KINASE CLK1	P49759	3.0
3dj6	SERINE/THREONINE KINASE 6	P97477	3.0
1j1b	GLYCOGEN SYNTHASE KINASE-3 BETA	P49841	3.0
3nyv	CALMODULIN-DOMAIN PROTEIN KINASE 1	Q9BJF5	3.0
4gv1	RAC-ALPHA SERINE/THREONINE-PROTEIN KINASE	P31749	3.0
4j7b	POLO-LIKE KINASE	Q6DRK7	3.0
3eyg	TYROSINE-PROTEIN KINASE	P23458	3.0
3nie	MAP2 KINASE	Q7KQK7	3.0
4wov	NON-RECEPTOR TYROSINE-PROTEIN KINASE TYK2	P29597	3.0
2vz6	CALCIUM CALMODULIN DEPENDENT PROTEIN KINASE TYPE II ALPHA CHAIN	Q9UQM7	3.0
4bf2	MITOGEN-ACTIVATED PROTEIN KINASE KINASE KINASE 5	Q99683	3.0
4cfe	5'-AMP-ACTIVATED PROTEIN KINASE CATALYTIC SUBUNIT ALPHA-2	P54646	3.0
3kk8	CALCIUM/CALMODULIN DEPENDENT PROTEIN KINASE II	O62305	3.0
2yab	DEATH-ASSOCIATED PROTEIN KINASE 2	Q8VDF3	3.0
3pg1	MITOGEN-ACTIVATED PROTEIN KINASE, PUTATIVE (MAP KINASE-LIKE PROTEIN)	Q4QHJ8	3.0
4ra4	PROTEIN KINASE C	P17252	3.0
2ozo	TYROSINE-PROTEIN KINASE ZAP-70	P43403	3.0
1ua2	CELL DIVISION PROTEIN KINASE 7	P50613	3.0
3bhh	CALCIUM/CALMODULIN-DEPENDENT PROTEIN KINASE TYPE II BETA	Q13554	3.0

4hzr	ACTIVATED CDC42 KINASE 1	Q07912	3.0
3qd2	EUKARYOTIC TRANSLATION INITIATION FACTOR 2-ALPHA KINASE 3	Q9Z2B5	3.0
4g3f	NF-KAPPA-BETA-INDUCING KINASE	Q9WUL6	3.0
4mwi	MIXED LINEAGE KINASE DOMAIN-LIKE PROTEIN	Q8NB16	3.0
3eqc	DUAL SPECIFICITY MITOGEN-ACTIVATED PROTEIN KINASE KINASE 1	Q02750	3.0
2w96	CELL DIVISION PROTEIN KINASE 4	P11802	3.0
2a2a	DEATH-ASSOCIATED PROTEIN KINASE 2	Q9UIK4	3.0
4oac	PROTEIN BRASSINOSTEROID INSENSITIVE 1	O22476	3.0
4myj	CGMP-DEPENDENT PROTEIN KINASE	Q8MMZ4	3.0
4an3	DUAL SPECIFICITY MITOGEN-ACTIVATED PROTEIN KINASE KINASE 1	Q02750	3.0
1zws	DAP-KINASE RELATED PROTEIN 1	Q9UIK4	3.0
3e3p	PROTEIN KINASE, PUTATIVE GLYCOGEN SYNTHASE KINASE	Q4QE15	3.0
4au8	CYCLIN-DEPENDENT KINASE 5	Q00535	3.0
4ks7	SERINE/THREONINE-PROTEIN KINASE PAK 6	Q9NQU5	3.0
1s9i	DUAL SPECIFICITY MITOGEN-ACTIVATED PROTEIN KINASE KINASE 2	P36507	2.9
4otw	RECEPTOR TYROSINE-PROTEIN KINASE ERBB-3	P21860	2.9
2jam	CALCIUM/CALMODULIN-DEPENDENT PROTEIN KINASE TYPE 1G	Q96NX5	2.9
3uc4	SERINE/THREONINE-PROTEIN KINASE SRK2E	Q940H6	2.9
2b9h	MITOGEN-ACTIVATED PROTEIN KINASE FUS3	P16892	2.9
2j7t	SERINE/THREONINE-PROTEIN KINASE 10	O94804	2.9
4uak	SERINE/THREONINE-PROTEIN KINASE MRCK BETA	Q9Y5S2	2.9
2pmn	SER/THR PROTEIN KINASE, PUTATIVE	Q7YTF7	2.9
4u6r	SERINE/THREONINE-PROTEIN KINASE/ENDORIBONUCLEASE IRE1	O75460	2.9
2buj	SERINE/THREONINE-PROTEIN KINASE 16	O75716	2.9
4rz7	CGMP-DEPENDENT PROTEIN KINASE, PUTATIVE	A5K0N4	2.9
3c0i	PERIPHERAL PLASMA MEMBRANE PROTEIN CASK	O14936	2.9
3fxz	SERINE/THREONINE-PROTEIN KINASE PAK 1	Q13153	2.9
1wak	SERINE/THREONINE-PROTEIN KINASE SPRK1	Q96SB4	2.9
4wno	SERINE/THREONINE-PROTEIN KINASE ULK1	O75385	2.9
2oza	MAP KINASE-ACTIVATED PROTEIN KINASE 2	P49137	2.9
1fvr	TYROSINE-PROTEIN KINASE TIE-2	Q02763	2.9
4nif	RIBOSOMAL PROTEIN S6 KINASE ALPHA-1	Q15418	2.9
4fvq	TYROSINE-PROTEIN KINASE JAK2	O60674	2.9
2x7g	UNCHARACTERIZED PROTEIN SRPK2	P78362	2.9
3h4j	SNF1-LIKE PROTEIN KINASE SSP2	O74536	2.9
2qr7	RIBOSOMAL PROTEIN S6 KINASE ALPHA-3	P18654	2.9
2rio	SERINE/THREONINE-PROTEIN KINASE/ENDORIBONUCLEASE IRE1	P32361	2.9
3mi9	CELL DIVISION PROTEIN KINASE 9	P50750	2.8
4nst	CYCLIN-DEPENDENT KINASE 12	Q9NYV4	2.8
2x4f	MYOSIN LIGHT CHAIN KINASE FAMILY MEMBER 4	Q86YV6	2.8
3kvw	DUAL SPECIFICITY TYROSINE-PHOSPHORYLATION-REGULATED KINASE 2	Q92630	2.8
4whz	MITOGEN-ACTIVATED PROTEIN KINASE 10	P53779	2.8

4y85	MITOGEN-ACTIVATED PROTEIN KINASE KINASE KINASE 8	P41279	2.8
4qtb	MITOGEN-ACTIVATED PROTEIN KINASE 3	P27361	2.8
2c47	CASEIN KINASE 1 GAMMA 2 ISOFORM	P78368	2.8
4bgq	CYCLIN-DEPENDENT KINASE-LIKE 5	O76039	2.8
3f3z	CALCIUM/CALMODULIN-DEPENDENT PROTEIN KINASE WITH A KINASE DOMAIN AND 4 CALMODULIN LIKE EF HANDS	Q5CYL9	2.8
3gni	STRAD ALPHA	Q7RTN6	2.8
2rku	SERINE/THREONINE-PROTEIN KINASE PLK1	P53350	2.8
4jlc	SERINE/THREONINE-PROTEIN KINASE TBK1	Q9WUN2	2.8
4c0t	LIKELY PROTEIN KINASE	Q5A3P6	2.8
3kn6	RIBOSOMAL PROTEIN S6 KINASE ALPHA-5	O75582	2.8
2f57	SERINE/THREONINE-PROTEIN KINASE PAK 7	Q9P286	2.8
4md7	CASEIN KINASE II SUBUNIT ALPHA	P68400	2.8
3llt	SERINE/THREONINE KINASE-1, PFLAMMER	Q8IL19	2.8
3war	CASEIN KINASE II SUBUNIT ALPHA	P68400	2.8
4e7w	GLYCOGEN SYNTHASE KINASE 3	N/A	2.8
4lqs	SERINE/THREONINE-PROTEIN KINASE CBK1	P53894	2.8
4l68	LEUCINE-RICH REPEAT PROTEIN KINASE-LIKE PROTEIN	Q9LSI9	2.8
4twc	CASEIN KINASE I ISOFORM DELTA	P48730	2.8
1nxk	MAP KINASE-ACTIVATED PROTEIN KINASE 2	P49137	2.8
3a8x	PROTEIN KINASE C IOTA TYPE	P41743	2.8
4rlk	CASEIN KINASE II SUBUNIT ALPHA	P28523	2.8
3fhr	MAP KINASE-ACTIVATED PROTEIN KINASE 3	Q16644	2.8
1vzo	RIBOSOMAL PROTEIN S6 KINASE ALPHA 5	O75582	2.7
4af3	AURORA KINASE B	Q96GD4	2.7
3ubd	RIBOSOMAL PROTEIN S6 KINASE ALPHA-3	P18654	2.7
3sv0	CASEIN KINASE I-LIKE	Q8LR51	2.7
4qpm	MITOTIC CHECKPOINT SERINE/THREONINE-PROTEIN KINASE BUB1	O43683	2.7
2izr	CASEIN KINASE I ISOFORM GAMMA-3	Q9Y6M4	2.7
2v62	SERINE/THREONINE-PROTEIN KINASE VRK2	Q86Y07	2.7
3alo	DUAL SPECIFICITY MITOGEN-ACTIVATED PROTEIN KINASE KINASE 4	P45985	2.7
4ic7	MITOGEN-ACTIVATED PROTEIN KINASE 7	Q13164	2.7
4im0	SERINE/THREONINE-PROTEIN KINASE TBK1	Q9UHD2	2.7
4eut	SERINE/THREONINE-PROTEIN KINASE TBK1	Q9UHD2	2.7
4yll	DUAL SPECIFICITY TYROSINE-PHOSPHORYLATION-REGULATED KINASE 1A	Q13627	2.7
4zzn	MITOGEN-ACTIVATED PROTEIN KINASE 1	P28482	2.7
4nfn	TAU-TUBULIN KINASE 1	Q5TCY1	2.7
4ix3	MSSTT7D PROTEIN	C1EBN1	2.7
4iir	SERINE/THREONINE-PROTEIN KINASE PRP4 HOMOLOG	Q13523	2.6
3ofm	CASEIN KINASE II SUBUNIT ALPHA'	P19784	2.6
4btk	TAU-TUBULIN KINASE 1	Q5TCY1	2.6
4i93	PROBABLE SERINE/THREONINE-PROTEIN KINASE AT5G41260	Q9FHD7	2.6

3m2w	MAP KINASE-ACTIVATED PROTEIN KINASE 2	P49137	2.6
3soa	CALCIUM/CALMODULIN-DEPENDENT PROTEIN KINASE TYPE II SUBUNIT ALPHA WITH A BETA 7 LINKER	Q9UQM7	2.6
4pf4	DEATH-ASSOCIATED PROTEIN KINASE 1	P53355	2.6
2hw6	MAP KINASE-INTERACTING SERINE/THREONINE-PROTEIN KINASE 1	Q9BUB5	2.6
4f9c	CELL DIVISION CYCLE 7-RELATED PROTEIN KINASE	O00311	2.6
4jr7	CASEIN KINASE II SUBUNIT ALPHA	P15790	2.6
3op5	SERINE/THREONINE-PROTEIN KINASE VRK1	Q99986	2.5
4jnw	TITIN	Q8WZ42	2.5
3cok	SERINE/THREONINE-PROTEIN KINASE PLK4	O00444	2.5
3zon	NON-RECEPTOR TYROSINE-PROTEIN KINASE TYK2	P29597	2.5
4o1p	RIBONUCLEASE L	A5H025	2.5
2cmw	CASEIN KINASE I ISOFORM GAMMA-1	Q9HCP0	2.5
1x8b	WEE1-LIKE PROTEIN KINASE	P30291	2.5
4oav	PROTEIN (RNASE L)	Q05823	2.4
4jrn	RHOPTRY KINASE FAMILY PROTEIN	Q2PAY2	2.4
4nt4	GILGAMESH, ISOFORM I	Q86NK8	2.3
4l00	TYROSINE-PROTEIN KINASE JAK1	P23458	2.3
3oht	P38A	A9UJZ9	2.3
1csn	CASEIN KINASE-1	P40233	2.1
4cvq	GLUTAMATE-PYRUVATE AMINOTRANSFERASE ALAA	P0A959	2.0

Table S2: Calculated docking and binding affinity (MMGBSA) scores of compounds for RIPK1 (PDB 4NEU) active site using different strategies. (A) The Glide SP docked poses. (B) Induced fit docking (IFD) poses. (C) Protein-ligand frames at every 10ns from the 100ns MD simulation. SD stands for standard deviation and N/A for not applicable as no valid pose was obtained.

A. Glide SP Docked complexes

Compound	Glide docking score	Glide gscore	MMGBSA dG Bind (kcal/mol)	MMGBSA dG Bind Coulomb (kcal/mol)
Cmpd8	-14.82	-15.02	-97.61	-30.13
GSK414	-13.33	-13.34	-59.60	-24.61
GSK157	-11.62	-11.63	-65.06	-18.40
AMG44	N/A	N/A	N/A	N/A

B. IFD docked complexes

Compound	IFD Docking score	IFD glide gscore	IFD Score	MMGBSA dG Bind (kcal/mol)	MMGBSA dG Bind Coulomb (kcal/mol)
Cmpd8	-14.65	-14.85	-607.51	-98.20	-33.78
GSK414	-15.21	-15.21	-604.49	-97.72	-33.33
GSK157	-14.86	-14.87	-605.99	-92.67	-21.11
AMG44	-15.51	-15.52	-602.96	-94.73	-5.86

C. IFD MD simulation trajectories

Compound	MMGBSA dG Bind Average (kcal/mol)	SD	MMGBSA dG Range	MMGBSA dG Bind Coulomb (kcal/mol)	SD
Cmpd8	-87.77	4.24	-95.40 to -80.62	-28.06	1.99
GSK414	-87.05	3.60	-93.25 to -81.18	-29.74	1.92
GSK157	-78.76	3.32	-87.31 to -74.79	-19.30	1.88
AMG44	-81.85	8.22	-95.49 to -70.53	-10.91	3.02

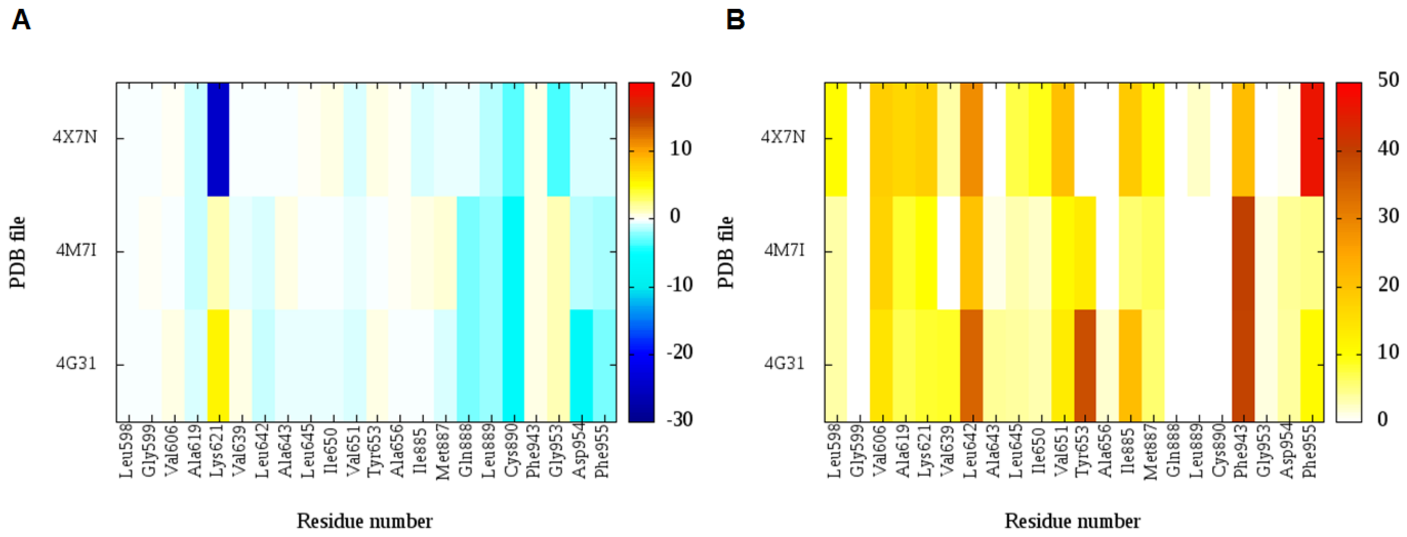

Figure S1. Per amino-acid interaction energy map for PERK co-crystal structures: crystal structure of GSK6414 (PDB code: 4G31), GSK6157 (PDB code: 4M7I) and AMG44 (PDB code: 4X7N). (A) Electrostatic energy values (kcal/mol). (B) Hydrophobic score (arbitrary units).

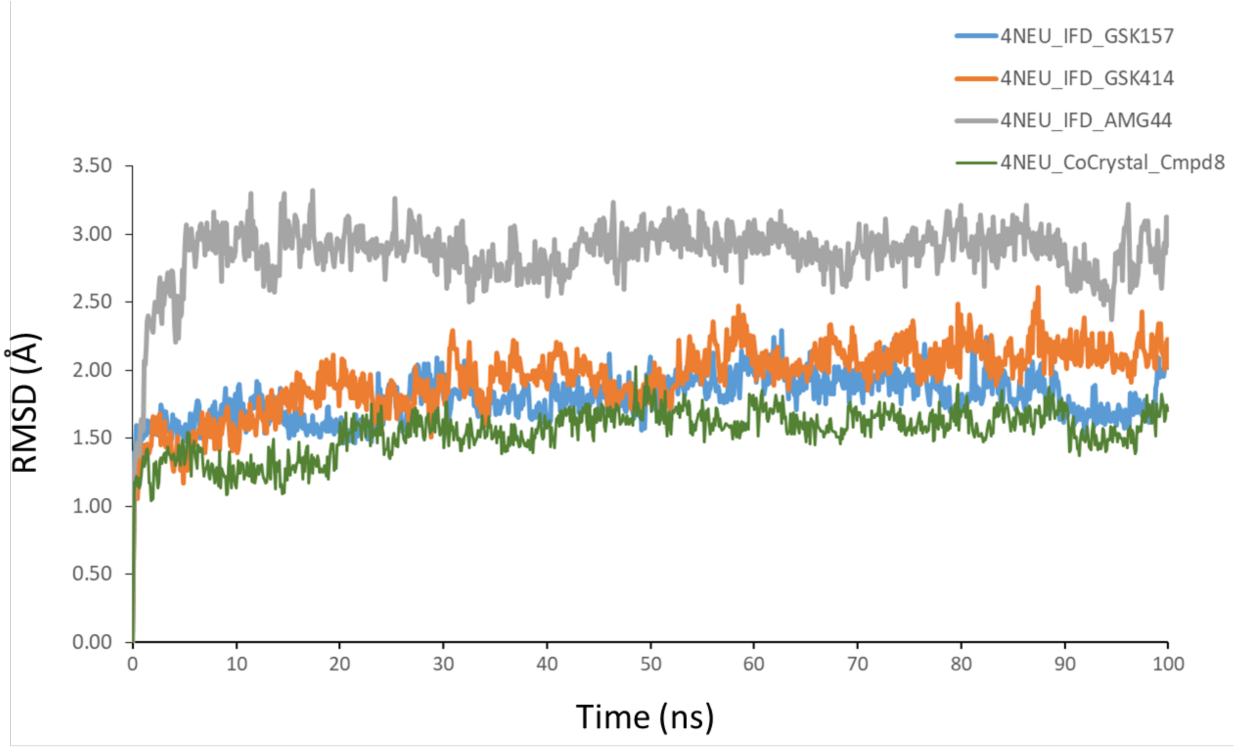

Figure S2. Active site dynamics of the RIPK1 complexes during 100ns MD simulations. RMSD of the active site residues is plotted as a function of MD simulation time.

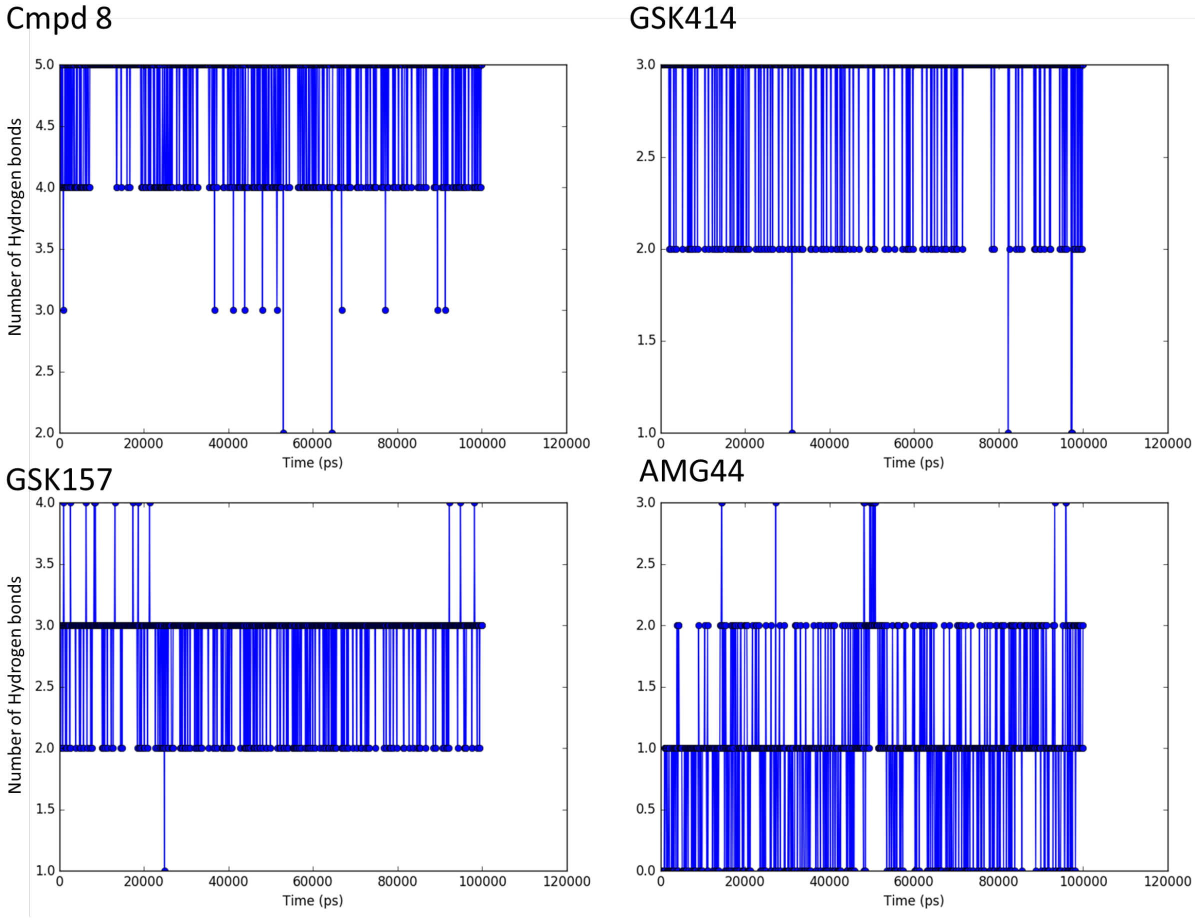

Figure S3. Comparison of the number of hydrogen bonds (H-bonds) formed by the compounds at the RIPK1 active site during the 100 ns MD simulation.

References

[1] J. Konc, T. Cesnik, J. T. Konc, M. Penca, D. Janezic, *J Chem Inf Model* **2012**, *52*, 604-612.
